# Supplementary material for: Kinetics of HIV-Specific CTL Responses Plays a Minimal Role in Determining HIV Escape Dynamics
Source: Front Immunol. 2018 Feb 8;9:140. doi: 10.3389/fimmu.2018.00140 (PMC5810297; doi:10.3389/fimmu.2018.00140)
Supplement: Supplementary file 1 [file Data_Sheet_1.PDF]

Supplementary Information

S1 Model derivation of viral escape from multiple CTL responses

Following the previous work [16], we use $m_{\mathbf{i}}$ to denote the density of variants denoted by a vector $\mathbf{i} = (i_1, i_2, ..., i_n)$, which is the index denoting the positions of n epitopes, and we define $i_j = 0$ if there is no mutation in the jth CTL epitope and $i_j = 1$ if there is a mutation leading to an escape from the jth CTL response.

We assume that a CTL response that recognizes the ith epitope of the virus kills the virus infected cells at rate k_i, and escaping from the ith CTL responses only at a rate μ_i leads to a viral replicative fitness cost c_i $(i = 1, ..., n)$. As shown in model (1) of viral escape from a single CTL response (see equation (1)), we denote the infection rate of variants $m_{\mathbf{i}}$ by $\beta_{\mathbf{i}}$ and variants $m_{\mathbf{i}}$ are produced by infected cells at rate $p_{\mathbf{i}}$ $(\mathbf{i} \in I)$. We assume that the wild-type has a higher (or equal) reproductive ratio, that is $\beta_{\mathbf{i}} p_{\mathbf{i}} \leq \beta_{(0,0,...,0)} p_{(0,0,...,0)}$ for all $\mathbf{i} \neq (0, 0, ..., 0)$ $(\mathbf{i} \in I)$.

Let $r = \frac{\beta_0 p_0}{c_v} T(t)$ (with $\beta_0 = \beta_{(0,0,...,0)}$ and $p_0 = p_{(0,0,...,0)}$) as the reproduction rate of wild-type virus, we use fitness cost c_i $(i = 1, ..., n)$ and r to express the replication rate of each escape variant. For simplicity, we neglect recombination and only allow single point mutation. To be consistent with the model of viral escape from a single CTL escape, we let β_i denote the rate at which variant $m_{(0,...,1,...,0)}$ (only i^{th} position equal to 1) infect cells, and p_i denote the production rate of variant $m_{(0,...,1,...,0)}$. Then the fitness cost c_i of $m_{(0,...,1,...,0)}$ can be written as $c_i = 1 - \frac{\beta_i p_i}{\beta_0 p_0}$ $(i = 1, ..., n)$. As for variants $m_{(i_1,...,i_n)}$ having two more mutations, we assume

$$\frac{\beta_{(i_1,...,i_n)} p_{(i_1,...,i_n)}}{\beta_0 p_0} = \prod_{\substack{j=1,...,n \\ i_j \neq 0}} \frac{\beta_j p_j}{\beta_0 p_0}. \qquad \text{(S1)}$$

This assumption means for variant having mutations at i^{th} and j^{th} epitopes, the normalized reproductive rate (by wild-type reproductive rate $\beta_0 p_0$) equals the product of normalized reproductive rates of variants, which only have one mutation at i^{th} or j^{th} epitope. For example, $\frac{\beta_{(1,1)} p_{(1,1)}}{\beta_0 p_0} = \frac{\beta_1 p_1}{\beta_0 p_0} \frac{\beta_2 p_2}{\beta_0 p_0}$ with $n = 2$. Under this assumption, the fitness cost $C_{(i_1,...,i_n)} = 1 - \frac{\beta_{(i_1,...,i_n)} p_{(i_1,...,i_n)}}{\beta_0 p_0}$ of

variant $m_{(i_1,...,i_n)}$ can be written as

$$C_{(i_1,...,i_n)} = 1 - \prod_{\substack{j=1,...,n \\ i_j \neq 0}} (1 - c_j). \tag{S2}$$

Assuming multiplicative fitness, the fitness cost of a variant $\mathbf{i} = (i_1, i_2, ..., i_n)$ is $C_{\mathbf{i}} = 1 - \prod_{j=1}^{n}(1 - c_j i_j)$. The death rate of the escape variant $\mathbf{i} = (i_1, i_2, ..., i_n)$ due to remaining CTL responses is given by $K_{\mathbf{i}} = \sum_{j=1}^{n} k_j(1 - i_j)$, where we assume that killing of infected cells by different CTL responses is additive.

We neglect recombination and backward mutation from mutant to wild-type in this modeling framework. More specifically, for two escape variants $m_{\mathbf{i}} = m_{(i_1,i_2,...,i_n)}$ and $m_{\mathbf{j}} = m_{(j_1,j_2,...,j_n)}$, we define the mutation rate $M_{\mathbf{i},\mathbf{j}}$ from $m_{\mathbf{i}}$ to $m_{\mathbf{j}}$ as μ_k, if and only if $m_{\mathbf{j}}$ has only one more mutation at position k than $m_{\mathbf{i}}$ and all other positions are exactly same. For example, when there are 3 CTL responses, the mutation rate from $m_{(1,0,0)}$ to $m_{(1,0,1)}$ is μ_3, and the mutation rate from $m_{(0,0,0)}$ to $m_{(1,0,1)}$ is 0.

Similar as equation (1), the dynamics of the wild-type and all escapes from CTL responses is given by

$$\frac{dm_{\mathbf{i}}(t)}{dt} = [r(1-C_{\mathbf{i}})(1-\sum_{\mathbf{j}\in I} M_{\mathbf{i},\mathbf{j}})-K_{\mathbf{i}}-\delta]m_{\mathbf{i}}(t)+\sum_{\mathbf{j}\in I} r(1-C_{\mathbf{j}})M_{\mathbf{j},\mathbf{i}}\frac{p_{\mathbf{i}}}{p_{\mathbf{j}}}m_{\mathbf{j}}(t),\ \mathbf{i} \in I. \tag{S3}$$

Here we adopt the simple assumption that escape mutants and wild-type viruses may differ from rates $\beta_{\mathbf{i}\in I}$ at which they infect cells, that is $p_0 = p_{\mathbf{i}}$ and $\beta_0 \geq \beta_{\mathbf{i}}$ ($\mathbf{i} \in I$ and $\mathbf{i} \neq (0, ..., 0)$). The system (S3) becomes

$$\frac{dm_{\mathbf{i}}(t)}{dt} = [r(1-C_{\mathbf{i}})(1-\sum_{\mathbf{j}\in I} M_{\mathbf{i},\mathbf{j}})-K_{\mathbf{i}}-\delta]m_{\mathbf{i}}(t)+\sum_{\mathbf{j}\in I} r(1-C_{\mathbf{j}})M_{\mathbf{j},\mathbf{i}}m_{\mathbf{j}}(t),\ \mathbf{i} \in I. \tag{S4}$$

We define $M(t) = \sum_{\mathbf{i}\in I} m_{\mathbf{i}}$ as the total density of all variants in the population, and $f_j(t)$ $(j = 1, ..., n)$ is the fraction of viral variants that have escaped recognition from the jth CTL response. Then

$$f_j(t) = \sum_{\mathbf{i}\in J} m_{\mathbf{i}}(t)/M(t),\ J = (i_1, ...i_j, ..., i_n) \text{ with } i_j = 1. \tag{S5}$$

For example, when $n = 2$, there are 3 types of escape variants $m_{(0,0)}$, $m_{(1,0)}$ and $m_{(1,1)}$ for "sequential" escape (model 2), and 4 types of escape variants $m_{(0,0)}$, $m_{(1,0)}$, $m_{(0,1)}$ and $m_{(1,1)}$ for "concurrent" escape (model 3).

Under all above assumptions, from system (S4), model 2 with $n = 2$ can be written as:

$$\begin{aligned}
\frac{dm_{(0,0)}(t)}{dt} =& [r(t)(1-\mu_1) - (\delta + k_1 + k_2)]m_{(0,0)}(t), \\
\frac{dm_{(1,0)}(t)}{dt} =& [r(t)(1-c_1)(1-\mu_2) - (\delta + k_2)]m_{(1,0)}(t) + \mu_1 r(t) m_{(0,0)}(t), \\
\frac{dm_{(1,1)}(t)}{dt} =& [r(t)(1-c_1)(1-c_2) - \delta]m_{(1,1)}(t) + r(t)(1-c_1)\mu_2 m_{(1,0)}(t).
\end{aligned} \tag{S6}$$

and

$$f_1(t) = \frac{m_{(1,0)}(t) + m_{(1,1)}(t)}{m_{(0,0)}(t) + m_{(1,0)}(t) + m_{(1,1)}(t)},$$
$$f_2(t) = \frac{m_{(1,1)}(t)}{m_{(0,0)}(t) + m_{(1,0)}(t) + m_{(1,1)}(t)}. \tag{S7}$$

Similarly, following system (S4), model 3 with $n = 2$ can be written as:

$$\begin{aligned}
\frac{dm_{(0,0)}(t)}{dt} =& [r(t)(1-\mu_1-\mu_2) - (\delta + k_1 + k_2)]m_{(0,0)}(t),\\
\frac{dm_{(1,0)}(t)}{dt} =& [r(t)(1-c_1)(1-\mu_2) - (\delta + k_2)]m_{(1,0)}(t) + \mu_1 r(t) m_{(0,0)}(t),\\
\frac{dm_{(0,1)}(t)}{dt} =& [r(t)(1-c_2)(1-\mu_1) - (\delta + k_1)]m_{(0,1)}(t) + \mu_2 r(t) m_{(0,0)}(t),\\
\frac{dm_{(1,1)}(t)}{dt} =& [r(t)(1-c_1)(1-c_2) - \delta]m_{(1,1)}(t) + r(t)(1-c_2)\mu_1 m_{(0,1)}(t)\\
&+ r(t)(1-c_1)\mu_2 m_{(1,0)}(t).
\end{aligned} \tag{S8}$$

and

$$f_1(t) = \frac{m_{(1,0)}(t) + m_{(1,1)}(t)}{m_{(0,0)}(t) + m_{(0,1)}(t) + m_{(1,0)}(t) + m_{(1,1)}(t)},$$
$$f_2(t) = \frac{m_{(0,1)}(t) + m_{(1,1)}(t)}{m_{(0,0)}(t) + m_{(0,1)}(t) + m_{(1,0)}(t) + m_{(1,1)}(t)}. \tag{S9}$$

S2 Examples of "sequential" and "concurrent" escapes for $n = 3$ epitopes/CTL responses

The difference between "sequential" escape (model 2) and "concurrent" escape (model 3) is the set of escape variants I. The set I has $n + 1$ elements for "sequential" escape model and 2^n elements for "concurrent" escape model for n epitope case. For the simple case $n = 3$, equations for all escape variants are

Model 2:

$$\begin{aligned}
\frac{dm_{(0,0,0)}(t)}{dt} =& [r(t)(1-\mu_1) - (\delta + k_1 + k_2 + k_3)]m_{(0,0,0)}(t),\\
\frac{dm_{(1,0,0)}(t)}{dt} =& [r(t)(1-c_1)(1-\mu_2) - (\delta + k_2 + k_3)]m_{(1,0,0)}(t) + \mu_1 r(t) m_{(0,0,0)}(t),\\
\frac{dm_{(1,1,0)}(t)}{dt} =& [r(t)(1-c_1)(1-c_2)(1-\mu_3) - (\delta + k_3)]m_{(1,1,0)}(t) + \mu_2 r(t)(1-c_1) m_{(1,0,0)}(t),\\
\frac{dm_{(1,1,1)}(t)}{dt} =& [r(t)(1-c_1)(1-c_2)(1-c_3) - \delta]m_{(1,1,1)}(t) + \mu_3 r(t)(1-c_1)(1-c_2) m_{(1,1,0)}(t),
\end{aligned} \tag{S10}$$

and

Model 3:

$$
\begin{aligned}
\frac{dm_{(0,0,0)}(t)}{dt} =&[r(t)(1-\mu_1-\mu_2-\mu_3)-(\delta+k_1+k_2+k_3)]m_{(0,0,0)}(t),\\
\frac{dm_{(1,0,0)}(t)}{dt} =&[r(t)(1-c_1)(1-\mu_2-\mu_3)-(\delta+k_2+k_3)]m_{(1,0,0)}(t)+\mu_1 r(t)m_{(0,0,0)}(t),\\
\frac{dm_{(0,1,0)}(t)}{dt} =&[r(t)(1-c_2)(1-\mu_1-\mu_3)-(\delta+k_1+k_3)]m_{(0,1,0)}(t)+\mu_2 r(t)m_{(0,0,0)}(t),\\
\frac{dm_{(0,0,1)}(t)}{dt} =&[r(t)(1-c_3)(1-\mu_1-\mu_2)-(\delta+k_1+k_2)]m_{(0,0,1)}(t)+\mu_3 r(t)m_{(0,0,0)}(t),\\
\frac{dm_{(1,1,0)}(t)}{dt} =&[r(t)(1-c_1)(1-c_2)(1-\mu_3)-(\delta+k_3)]m_{(1,1,0)}(t)+\mu_1(1-c_2)r(t)m_{(0,1,0)}(t)\\
&+\mu_2(1-c_1)r(t)m_{(1,0,0)}(t),\\
\frac{dm_{(1,0,1)}(t)}{dt} =&[r(t)(1-c_1)(1-c_3)(1-\mu_2)-(\delta+k_2)]m_{(1,0,1)}(t)+\mu_1(1-c_3)r(t)m_{(0,0,1)}(t)\\
&+\mu_3(1-c_1)r(t)m_{(1,0,0)}(t),\\
\frac{dm_{(0,1,1)}(t)}{dt} =&[r(t)(1-c_2)(1-c_3)(1-\mu_1)-(\delta+k_1)]m_{(0,1,1)}(t)+\mu_2(1-c_3)r(t)m_{(0,0,1)}(t)\\
&+\mu_3(1-c_2)r(t)m_{(0,1,0)}(t),\\
\frac{dm_{(1,1,1)}(t)}{dt} =&[r(t)(1-c_1)(1-c_2)(1-c_3)-\delta]m_{(1,1,1)}(t)+\mu_1(1-c_2)(1-c_3)r(t)m_{(0,1,1)}(t)\\
&+\mu_2(1-c_1)(1-c_3)r(t)m_{(1,0,1)}(t)+\mu_3(1-c_1)(1-c_2)r(t)m_{(1,1,0)}(t).
\end{aligned}
\tag{S11}
$$

S3 Additional results of the analysis

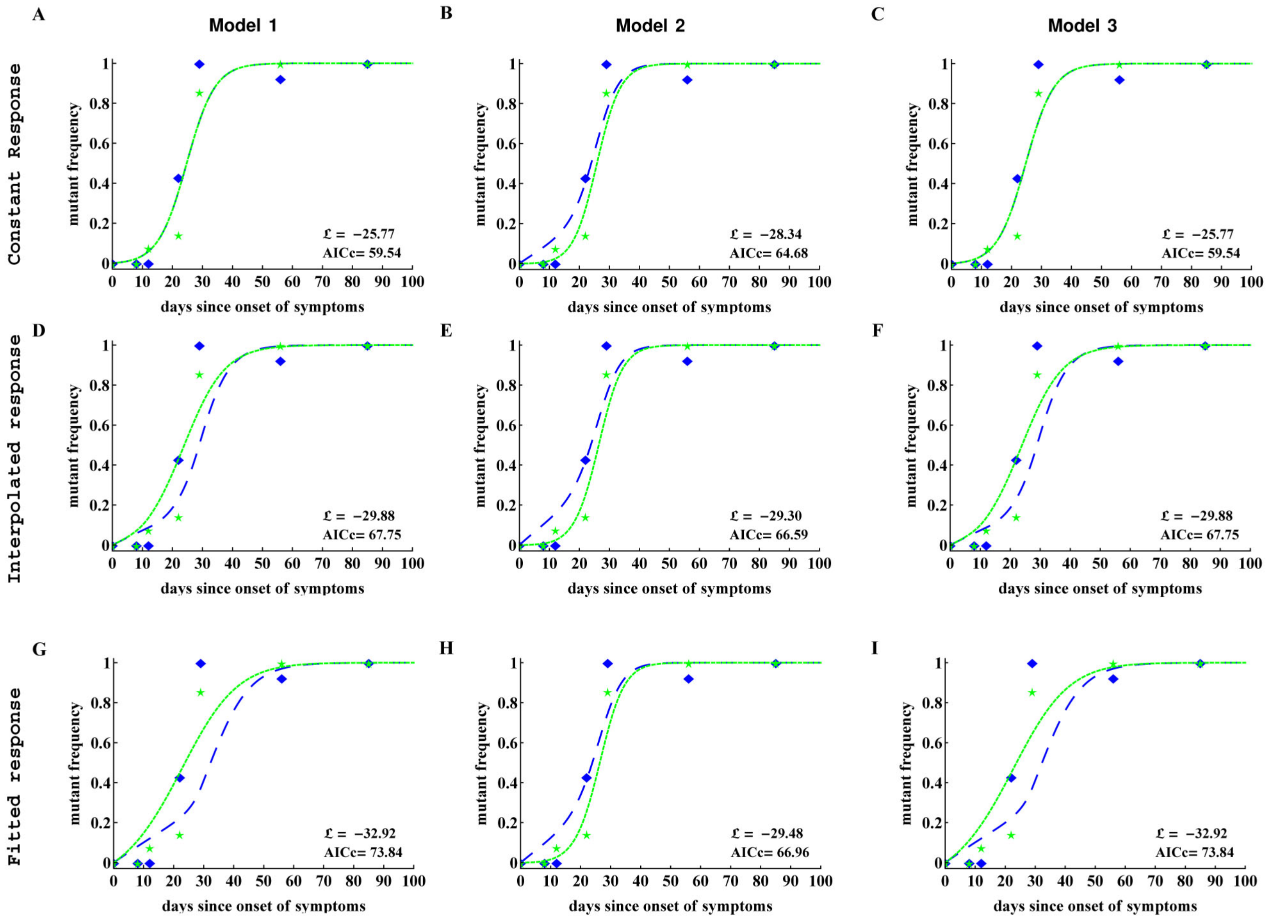

Figure S1 : Mathematical model accurately explains kinetics of HIV escape from CTL response when assuming equal mutation rates ($\mu_1 = \mu_2$) for data from patient CH159. We fit the three mathematical models (models 1, 2, and 3) to experimental data using likelihood approach outlined in the Materials and Methods section assuming $\mu_1 = \mu_2$. Three different models for the CTL response dynamics were assumed: constant input, interpolated input and fitted input. Models with response input did not improve the quality of the model fit to data. The best fit was provided by the models 1&3 with constant response. Estimated parameter values are given in Table S1. Notations for data points and lines are identical to those given in Figure 4 in the main text.

	peptide	model 1		model 2		model 3	
constant response		mutation rate (μ_i, i=1,2)	killing rate (k_i, i=1,2)	mutation rate (μ_i, i=1,2)	killing rate (k_i, i=1,2)	mutation rate (μ_i, i=1,2)	killing rate (k_i, i=1,2)
	Rev 65-82	7.55×10^{-4}	0.21	*6.75×10^{-3}*	1.86×10^{-13}	7.55×10^{-4}	0.21
	Nef 177-194		0.21		0.25		0.21
		$\mathcal{L} = -\mathbf{25.77}$, $AICc = \mathbf{59.54}$		$\mathcal{L} = -28.34$, $AICc = 64.68$		$\mathcal{L} = -\mathbf{25.77}$, $AICc = \mathbf{59.54}$	
interpolated response		mutation rate (μ_i, i=1,2)	killing rate (k'_i, i=1,2)	mutation rate (μ_i, i=1,2)	killing rate (k'_i, i=1,2)	mutation rate (μ_i, i=1,2)	killing rate (k'_i, i=1,2)
	Rev 65-82	*5.98×10^{-3}*	3.07×10^{-3}	*8.88×10^{-3}*	3.69×10^{-12}	*4.98×10^{-3}*	3.07×10^{-3}
	Nef 177-194		1.51×10^{-3}		2.74×10^{-3}		1.51×10^{-3}
		$\mathcal{L} = -29.88$, $AICc = 67.75$		$\mathcal{L} = -29.30$, $AICc = 66.59$		$\mathcal{L} = -29.88$, $AICc = 67.75$	
fitted response		mutation rate (μ_i, i=1,2)	killing rate (k'_i, i=1,2)	mutation rate (μ_i, i=1,2)	killing rate (k'_i, i=1,2)	mutation rate (μ_i, i=1,2)	killing rate (k'_i, i=1,2)
	Rev 65-82	*8.73×10^{-3}*	1.98×10^{-3}	*8.19×10^{-3}*	6.32×10^{-9}	*8.72×10^{-3}*	1.98×10^{-3}
	Nef 177-194		9.22×10^{-4}		2.69×10^{-3}		9.23×10^{-4}
		$\mathcal{L} = -34.92$, $AICc = 77.85$		$\mathcal{L} = -29.48$, $AICc = 66.97$		$\mathcal{L} = -34.92$, $AICc = 77.85$	

Table S1 : Best fit parameters of three models (models 1, 2 and 3) fitted to experimental data on HIV escape in patient CH159 assuming identical mutation rates ($\mu_1 = \mu_2$). Model fits are shown in Figure S1. $\mathcal{L}$ and AICc give the log-likelihood score and the correlated Akaike information criterion value, respectively. Best $\mathcal{L}$ (maximum) and AICc (minimum) scores are shown in bold. Mutation rates which exceed a theoretically assumed maximum value of 10^{-3} are shown in italics.

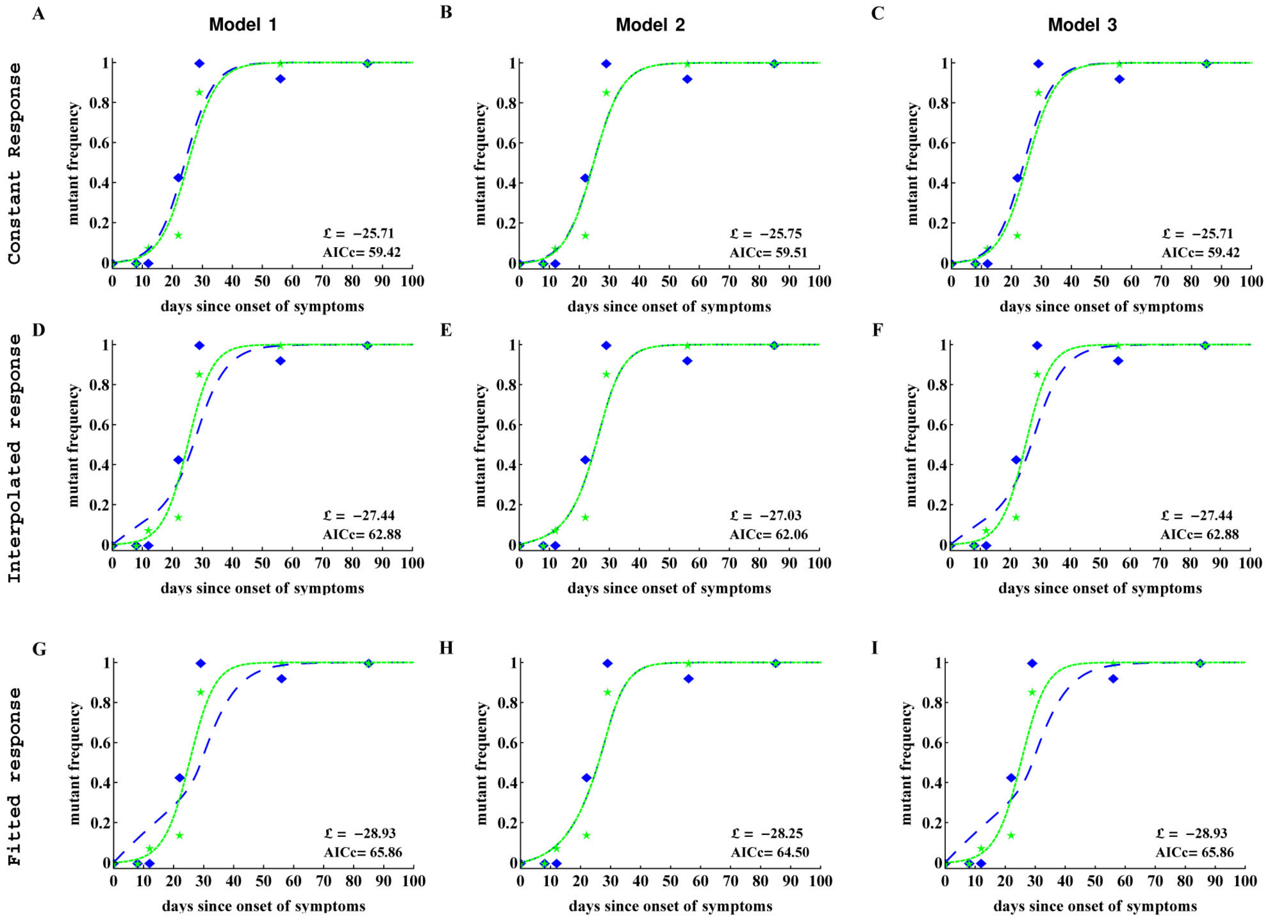

Figure S2 : Killing rates of CTL responses specific to different epitopes may be similar. We fit three different models to experimental data from patient CH159 assuming identical CTL killing rates ($k_1 = k_2$) with different CTL response dynamics (constant input, interpolated input and fitted input). Such constrain did not reduce the quality of the model fit to data as judged by AIC. Parameter estimates are given in Table S2. Notations for data points and lines are identical to those given in Figure 4 in the main text.

	peptide	model 1		model 2		model 3	
constant response		mutation rate (μ_i, i=1,2)	killing rate (k_i, i=1,2)	mutation rate (μ_i, i=1,2)	killing rate (k_i, i=1,2)	mutation rate (μ_i, i=1,2)	killing rate (k_i, i=1,2)
	Rev 65-82	8.39×10^{-4}	0.21	9.75×10^{-4}	0.10	8.39×10^{-4}	0.21
	Nef 177-194	6.61×10^{-4}		*0.30*		6.61×10^{-4}	
		$\mathcal{L} = -\mathbf{25.71}$, $AICc$= **59.42**		$\mathcal{L} = -25.75$, $AICc$= 59.51		$\mathcal{L} = -\mathbf{25.71}$, $AICc$= **59.42**	
interpolated response		mutation rate (μ_i, i=1,2)	killing rate (k'_i, i=1,2)	mutation rate (μ_i, i=1,2)	killing rate (k'_i, i=1,2)	mutation rate (μ_i, i=1,2)	killing rate (k'_i, i=1,2)
	Rev 65-82	$\mathit{7.71 \times 10^{-3}}$	2.75×10^{-3}	$\mathit{2.60 \times 10^{-3}}$	1.50×10^{-3}	$\mathit{7.70 \times 10^{-3}}$	2.75×10^{-3}
	Nef 177-194	8.91×10^{-4}		*13282.59*		8.85×10^{-4}	
		$\mathcal{L} = -27.44$, $AICc$= 62.88		$\mathcal{L} = -27.03$, $AICc$= 62.06		$\mathcal{L} = -27.44$, $AICc$= 62.88	
fitted response		mutation rate (μ_i, i=1,2)	killing rate (k'_i, i=1,2)	mutation rate (μ_i, i=1,2)	killing rate (k'_i, i=1,2)	mutation rate (μ_i, i=1,2)	killing rate (k'_i, i=1,2)
	Rev 65-82	$\mathit{1.26 \times 10^{-2}}$	2.35×10^{-3}	$\mathit{2.34 \times 10^{-3}}$	1.69×10^{-3}	$\mathit{1.26 \times 10^{-2}}$	2.35×10^{-3}
	Nef 177-194	9.34×10^{-4}		*7186.74*		9.33×10^{-4}	
		$\mathcal{L} = -30.53$, $AICc$= 69.06		$\mathcal{L} = -29.33$, $AICc$= 66.66		$\mathcal{L} = -30.53$, $AICc$= 69.06	

Table S2 : Best fit parameters of three models (models 1, 2 and 3) fitted to experimental data on HIV escape in patient CH159 assuming identical killing rates ($k_1 = k_2$). Model fits are shown in Figure S2. High (perhaps unrealistic) mutation rates are highlighted in italic. $\mathcal{L}$ and AICc give the log-likelihood score and the correlated Akaike information criterion value, respectively. Best $\mathcal{L}$ (maximum) and AICc (minimum) scores are shown in bold.

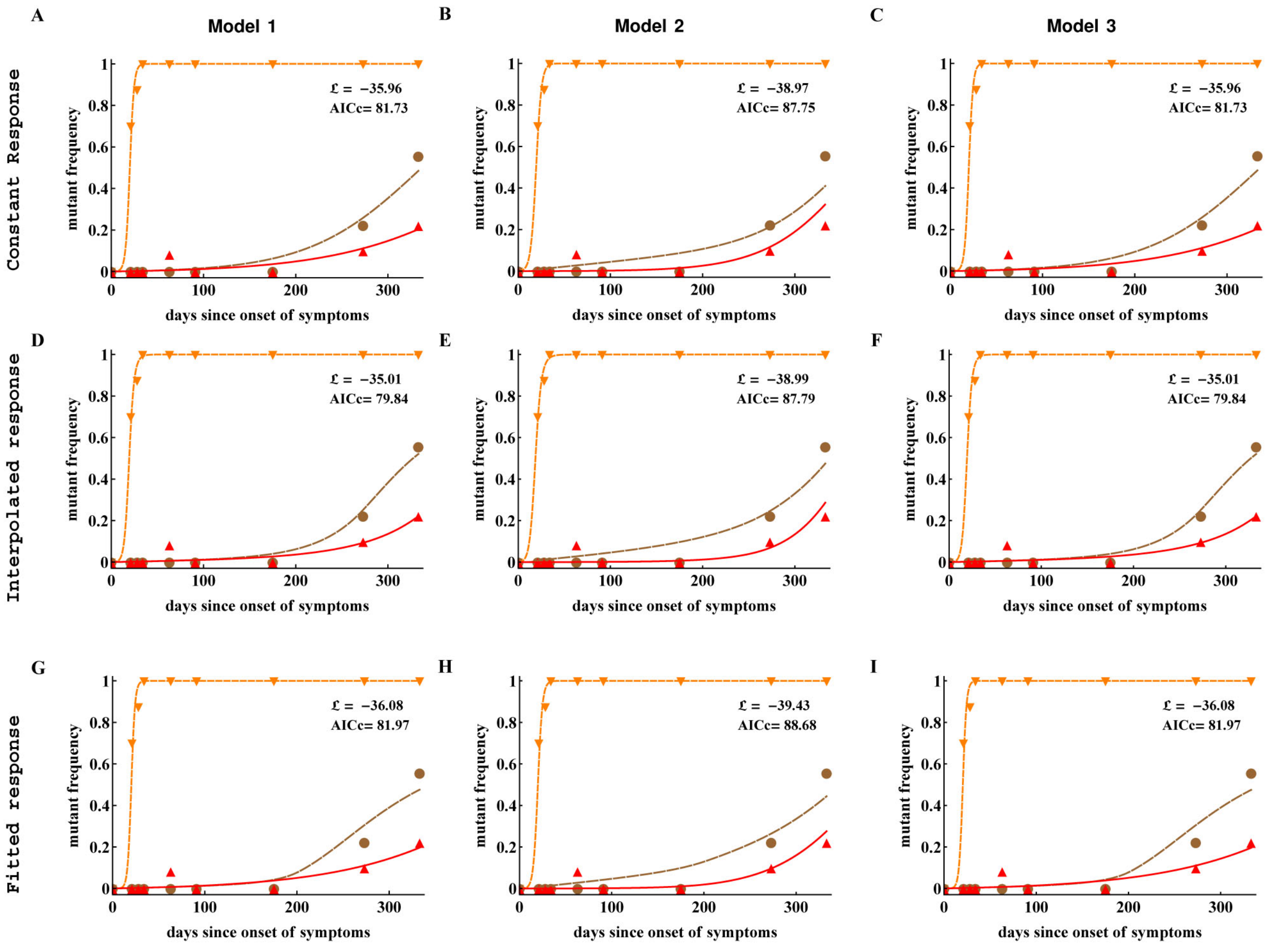

Figure S3 : Mathematical models accurately explain kinetics of HIV escape from CTL response when assuming equal mutation rates ($\mu_1 = \mu_2 = \mu_3$) for data from patient CH131. We fit the three mathematical models (models 1, 2, and 3) to experimental data using likelihood approach outlined in the Materials and Methods section assuming $\mu_1 = \mu_2 = \mu_3$. Three different models for the CTL response dynamics were assumed: no input, interpolated input and fitted input. Best model fit was provided by the models 1&3 with interpolated response input. Estimated parameter values are given in Table S3. Notations for data points and lines are identical to those given in Figure 6 in the main text.

	peptide	model 1		model 2		model 3	
Constant response		mutation rate (μ_i, $i = 1,2,3$)	killing rate (k_i, $i = 1,2,3$)	mutation rate (μ_i, $i = 1,2,3$)	killing rate (k_i, $i = 1,2,3$)	mutation rate (μ_i, $i = 1,2,3$)	killing rate (k_i, $i = 1,2,3$)
	Nef 64-74	4.34×10^{-5}	0.44	3.05×10^{-4}	0.34	4.34×10^{-5}	0.44
	Env 709-726		0.016		1.44×10^{-10}		0.016
	Gag 156-173		0.011		0.021		0.011
		$\mathcal{L} = -35.96$, $AICc = 81.73$		$\mathcal{L} = -38.97$, $AICc = 87.75$		$\mathcal{L} = -35.96$, $AICc = 81.73$	
interpolated response		mutation rate (μ_i, $i = 1,2,3$)	killing rate (k'_i, $i = 1,2,3$)	mutation rate (μ_i, $i = 1,2,3$)	killing rate (k'_i, $i = 1,2,3$)	mutation rate (μ_i, $i = 1,2,3$)	killing rate (k'_i, $i = 1,2,3$)
	Nef 64-74	6.22×10^{-5}	2.53×10^{-4}	3.00×10^{-4}	2.07×10^{-4}	6.22×10^{-5}	2.53×10^{-4}
	Env 709-726		1.87×10^{-5}		5.33×10^{-6}		1.87×10^{-5}
	Gag 156-173		8.73×10^{-6}		1.24×10^{-5}		8.73×10^{-6}
		$\mathcal{L} = \mathbf{-35.01}$, $AICc = \mathbf{79.84}$		$\mathcal{L} = -38.99$, $AICc = 87.79$		$\mathcal{L} = \mathbf{-35.01}$, $AICc = \mathbf{79.84}$	
fitted response		mutation rate (μ_i, $i = 1,2,3$)	killing rate (k'_i, $i = 1,2,3$)	mutation rate (μ_i, $i = 1,2,3$)	killing rate (k'_i, $i = 1,2,3$)	mutation rate (μ_i, $i = 1,2,3$)	killing rate (k'_i, $i = 1,2,3$)
	Nef 64-74	7.48×10^{-5}	6.72×10^{-4}	3.21×10^{-4}	5.41×10^{-4}	7.65×10^{-5}	6.70×10^{-4}
	Env 709-726		1.19×10^{-5}		2.88×10^{-6}		1.18×10^{-5}
	Gag 156-173		5.82×10^{-6}		1.00×10^{-5}		5.74×10^{-6}
		$\mathcal{L} = -36.08$, $AICc = 81.97$		$\mathcal{L} = -39.43$, $AICc = 88.68$		$\mathcal{L} = -36.08$, $AICc = 81.97$	

Table S3 : Best fit parameter values found by fitting different mathematical models (models 1, 2 and 3) to experimental data in patient CH131 assuming identical mutation rates ($\mu_1 = \mu_2 = \mu_3$). Model fits are shown in Figure S3. $\mathcal{L}$ and AICc give the log-likelihood score and the correlated Akaike information criterion value, respectively. Best $\mathcal{L}$ (maximum) and AICc (minimum) scores are bolded in the table.

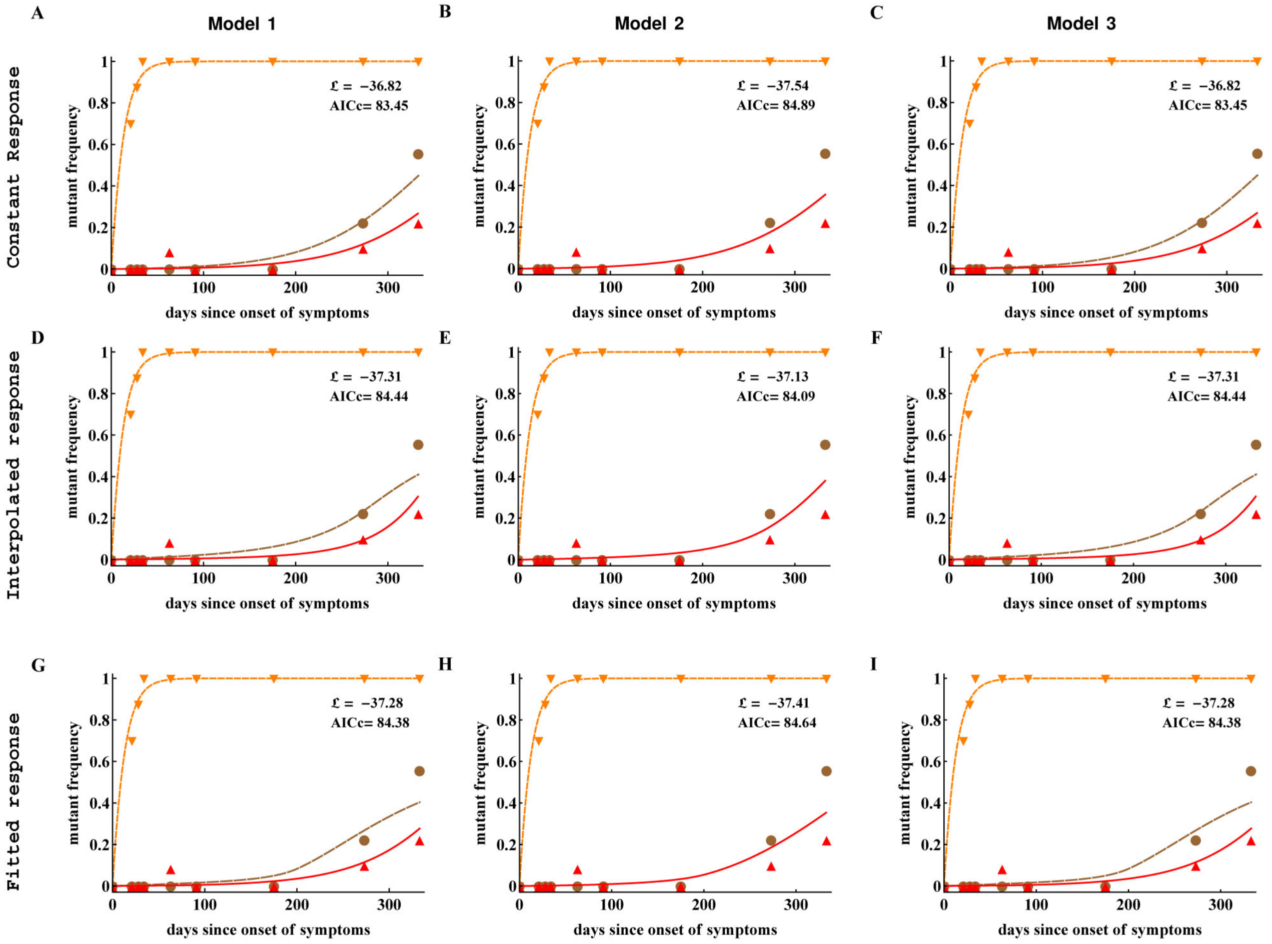

Figure S4 : No evidence for difference in CTL killing rates for data on HIV escape in patient CH131. We fit different mathematical models (models 1, 2, or 3) to experimental data from patient CH131 assuming equal killing rates ($k_1 = k_2 = k_3$). The best fit is given by models 1&3 with interpolated response input. Best fit parameter values are given in Table S4. Notations for data points and lines are identical to those given in Figure 6 in the main text.

	peptide	model 1		model 2		model 3	
		mutation rate (μ_i, $i = 1, 2, 3$)	killing rate (k_i, $i = 1, 2, 3$)	mutation rate (μ_i, $i = 1, 2, 3$)	killing rate (k_i, $i = 1, 2, 3$)	mutation rate (μ_i, $i = 1, 2, 3$)	killing rate (k_i, $i = 1, 2, 3$)
Constant response	Nef 64-74	*0.047*	0.017	*0.051*	7.88×10^{-3}	*0.047*	0.016
	Env 709-726	3.68×10^{-5}		3.60×10^{-5}		3.69×10^{-5}	
	Gag 156-173	1.67×10^{-5}		*81454.11*		1.68×10^{-5}	
		$\mathcal{L} = -\mathbf{36.82}$, $AICc$= **83.45**		$\mathcal{L} = -37.54$, $AICc$= 84.89		$\mathcal{L} = -\mathbf{36.82}$, $AICc$= **83.45**	
		mutation rate (μ_i, $i = 1, 2, 3$)	killing rate (k'_i, $i = 1, 2, 3$)	mutation rate (μ_i, $i = 1, 2, 3$)	killing rate (k'_i, $i = 1, 2, 3$)	mutation rate (μ_i, $i = 1, 2, 3$)	killing rate (k'_i, $i = 1, 2, 3$)
interpolated response	Nef 64-74	*0.046*	1.28×10^{-5}	*0.050*	6.65×10^{-6}	*0.046*	1.28×10^{-5}
	Env 709-726	1.31×10^{-4}		6.82×10^{-5}		1.31×10^{-4}	
	Gag 156-173	3.09×10^{-5}		*2.39×10^{7}*		3.07×10^{-5}	
		$\mathcal{L} = -37.31$, $AICc$= 84.44		$\mathcal{L} = -37.13$, $AICc$= 84.09		$\mathcal{L} = -37.31$, $AICc$= 84.44	
		mutation rate (μ_i, $i = 1, 2, 3$)	killing rate (k'_i, $i = 1, 2, 3$)	mutation rate (μ_i, $i = 1, 2, 3$)	killing rate (k'_i, $i = 1, 2, 3$)	mutation rate (μ_i, $i = 1, 2, 3$)	killing rate (k'_i, $i = 1, 2, 3$)
fitted response	Nef 64-74	*0.051*	9.79×10^{-6}	*0.053*	5.07×10^{-6}	*0.051*	9.78×10^{-6}
	Env 709-726	1.02×10^{-4}		5.51×10^{-5}		1.02×10^{-4}	
	Gag 156-173	2.67×10^{-5}		*2.73×10^{7}*		2.67×10^{-5}	
		$\mathcal{L} = -37.28$, $AICc$= 84.38		$\mathcal{L} = -37.41$, $AICc$= 84.64		$\mathcal{L} = -37.28$, $AICc$= 84.38	

Table S4 : Best fit parameter values found by fitting different mathematical models (models 1, 2 and 3) to experimental data in patient CH131 assuming identical killing rates ($k_1 = k_2 = k_3$). Model fits are shown in Figure S3. $\mathcal{L}$ and AICc give the log-likelihood score and the correlated Akaike information criterion value, respectively. Best $\mathcal{L}$ (maximum) and AICc (minimum) scores are bolded in the table. High (perhaps unrealistic) mutation rates are highlighted in italic.
